# Knowledge, attitude, and practices towards cutaneous leishmaniasis in referral cases with cutaneous lesions: A cross-sectional survey in remote districts of southern Khyber Pakhtunkhwa, Pakistan

**DOI:** 10.1371/journal.pone.0268801

**Published:** 2022-05-26

**Authors:** Salman Ahmad, Muhammad Kashif Obaid, Muhammad Taimur, Huma Shaheen, Shahid Niaz Khan, Sadaf Niaz, Rehman Ali, Sumbal Haleem

**Affiliations:** 1 Faculty of Biological Sciences, Department of Zoology, Kohat University of Science and Technology, Khyber Pakhtunkhwa, Pakistan; 2 Department of Zoology, Abdul Wali Khan University Garden Campus Mardan, Khyber Pakhtunkhwa, Pakistan; 3 Government Girls Degree College Tarkha, Nowshera, Pakistan; Iran University of Medical Sciences, ISLAMIC REPUBLIC OF IRAN

## Abstract

**Background:**

Cutaneous leishmaniasis is a neglected tropical disease caused by *Leishmania* spp. and transmitted by female sandflies. Terrorism and counter-insurgency military operations in Federally Administered Tribal Areas (FATA) lead to a large-scale migration of internally displaced persons (IDPs) in Khyber Pakhtunkhwa and thus, new outbreaks of several infectious diseases such as cutaneous leishmaniasis occurred. This study intended to find the prevalence of cutaneous leishmaniasis in people with cutaneous lesions suspected of having cutaneous leishmaniasis in four remote districts of Khyber Pakhtunkhwa and to assess the participant’s knowledge, attitude, and practices about the infection and its control.

**Methods:**

A cross-sectional study was carried out in four remote districts of Khyber Pakhtunkhwa including Karak, Lakki Marwat, Tank, and Dera Ismail Khan (D. I. Khan) and a total of 1,674 participants were recruited using a convenience sampling technique.

**Results:**

The prevalence of cutaneous leishmaniasis among the participants with cutaneous lesions was 50.4% and the infection was comparatively more prevalent in district Karak. Among participants, 56.8% were male and mostly, 53.8% were under the age of 16 years with 52.8% living in kutcha houses and were from rural areas. Multiple skin lesions were more common, and the face was frequently affected body part. The ratio of participants with lesions older than a month was higher and the majority confronted infections with blood protozoan parasites for the first time. Most participants were unaware of the signs/symptoms of the disease, basic knowledge of the vectors, anthroponotic spread, preventive measures, secondary infections, and reservoir hosts. The use of wood/animal dung as fuel, closeness with reservoir animals, and no use of insect repellents were some of the notable risk factors.

**Conclusion:**

Cutaneous leishmaniasis is highly prevalent in the study area and a very low level of awareness was reported among the participants. This study necessitates the planning and execution of regulations and preventive programs, public health education, awareness campaigns, and disease management practices to overcome future incidence of cutaneous leishmaniasis.

## Introduction

Leishmaniasis is a vector-borne disease caused by obligate intracellular protozoan parasites of the genus *Leishmania* [1] and transmitted by the bite of female sandflies of *Phlebotomus* and *Lutzomyia* species [2]. Leishmaniasis is a complex global disease that is widespread and is endemic in Asia, Africa, the Americas, and the Mediterranean region. It is one of the world’s most neglected poverty‐related diseases, affecting the poorest people in developing countries and is linked with malnutrition, immune system deficiency, migration, inadequate education, illiteracy, gender inequality, and a shortage of services [3]. Different therapeutic options include chemical (antimonial compounds), physical (cryotherapy, surgery, and thermotherapy) [4], and even herbal treatment modalities [5] are usually used to reduce the complications and side effects of leishmaniasis.

Different human activities such as wars, deforestation, and agricultural practices greatly influenced the habitat and distribution of sandflies leading to the resurgence of cutaneous leishmaniasis [6]. Other climatic factors including rainfall, global warming, humidity, and ambient temperature have serious impacts on the disease distribution and affect the vectors, hosts, and *Leishmania* spp. in various ways [7]. Poor and unhygienic sanitary conditions, uncovered water pots, cracks in walls, and houses assembled of grasses may favour the breeding of sandflies. Furthermore, the presence of a large number of people in a small area can attract sandflies, and migration due to socioeconomic factors may also contribute to the development of other risk factors for cutaneous leishmaniasis [7].

Pakistan is home to several infectious zoonoses [8,9], vector-borne diseases [10,11], and vectors [12]. However, cutaneous leishmaniasis greatly affected the people of Pakistan, specifically those of Khyber Pakhtunkhwa province, and several studies reported the disease in southern Khyber Pakhtunkhwa [13–17]. The latest epidemic resulted in almost 28,000 cases of leishmaniasis in Khyber Pakhtunkhwa province [18]. The infection in Khyber Pakhtunkhwa, which is caused by *L*. *tropica* and *L*. *major*, is mostly spread because of the influx of several million refugees into this province [15]. Various endemic foci have been registered regularly from various tribal agencies [15,19] and other settled districts of Khyber Pakhtunkhwa, including Dera Ismail Khan [20], Kohat [13,14], Karak [16,21], Dir [22], Sawat [23], Charsadda [24], and Malakand [25] among others.

Knowledge, attitude, and practices (KAP) surveys are very important for assessing the awareness and perception of a disease in a local population. Only a few reports are available regarding the knowledge and perception of the disease in Pakistan [26–28]. These surveys reported a moderate to low level of knowledge about the disease transmission, risk factors, and vectors among the population [26,27]. Alarmingly, in some areas, the practice and attitudes of the participants were not satisfactory as close to half of the population did adopt any control method [28]. While in other areas, most of the respondents showed a positive attitude towards disease seriousness and believed in treating the infection with modern medicines [27]. The prevalence of the disease is regularly increasing in southern areas of Khyber Pakhtunkhwa and is becoming one of the major health problems. In Pakistan, all previous KAP based studies have reported the knowledge and awareness of the general population. However, this study is specifically intended to report the prevalence, knowledge, attitude, and practices of the disease among people with cutaneous lesions suspected of having cutaneous leishmaniasis in four remote districts, namely, Tank, Karak, Dera Ismail Khan, and Lakki Marwat of Khyber Pakhtunkhwa. The study area is important because millions of IDPs migrated to camps, during military operations against terrorism in the South and North Waziristan Agency by Pakistan armed forces [29], located in these districts. The IDPs had migrated from hyperendemic areas (Pak-Afghan border) which may result in high cutaneous leishmaniasis prevalence in Khyber Pakhtunkhwa [14]. Therefore, it is indispensable to explore the prevalence of the infection in people with cutaneous lesions suspected of having cutaneous leishmaniasis in the above-mentioned remote districts and to assess the participant’s knowledge, attitude, and practices about the disease and its control.

## Materials and methods

### Ethical consideration and consent to participate

Ethical approval for the study was obtained from the Research Ethical Committee of Kohat University of Science & Technology (KUST). Before sampling, participants/guardians were briefed about the proposed study, and written informed consent was obtained. All participants were assured that the information will not be disclosed to any person and will be used for research purposes only. All methods were performed following the relevant guidelines and regulations.

### Study area

**Fig 1:**
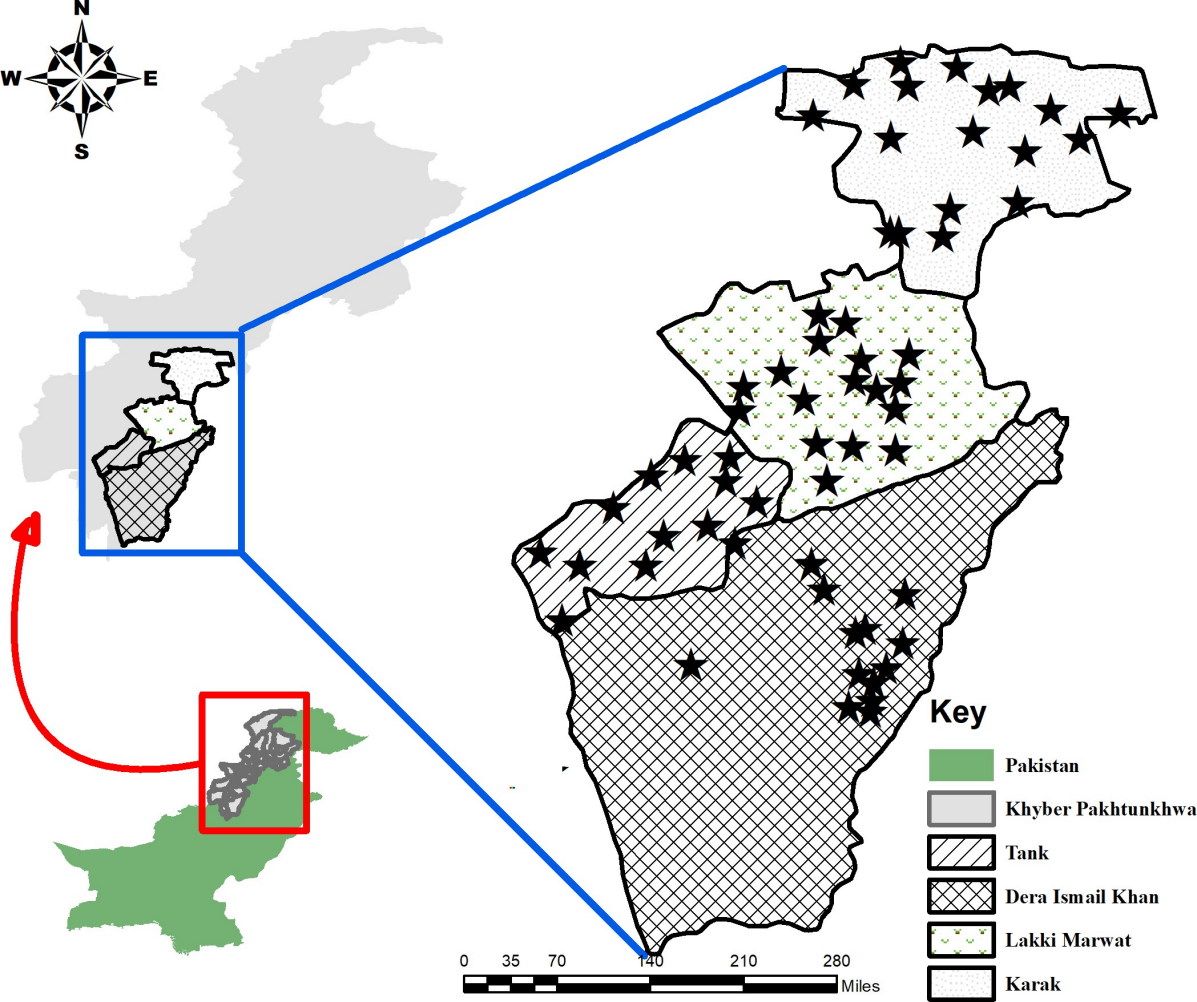
Map of the study area showing four districts of the southern Khyber Pakhtunkhwa, Pakistan (Drawn using the software “ArcGIS” (https://desktop.arcgis.com/en/).

Four districts of the southern Khyber Pakhtunkhwa including Karak (33.110786° N, 71.091346° E), Lakki Marwat (32.608018° N, 70.906081° E), Tank (32.216056° N, 70.389512° E), and Dera Ismail Khan (31.862683° N, 70.902157° E) were selected for this study. Khyber Pakhtunkhwa is one of the four provinces of Pakistan comprised of an area of 74, 521 km^2^ and a total population of 35 million (https://www.pbs.gov.pk/content/population-census). Khyber Pakhtunkhwa is considered endemic for cutaneous leishmaniasis and the four districts were purposively selected based on the outbreaks which have been previously reported [16,18,30,31] **(Fig 1)**. So far, no published clinical reports/studies have been found on cutaneous leishmaniasis in district Tank, however, the district is bounded to the west by North Waziristan Agency (NWA) which is considered endemic for this infection [15]. The inhabitants of Khyber Pakhtunkhwa are mostly Pashtoon/Pathan and the major first language is Pashto with slight differences in the accent from area to area. Urdu (native language) and English (partly) are also spoken and understood by the inhabitants.

### Study population and sampling

**Fig 2:**
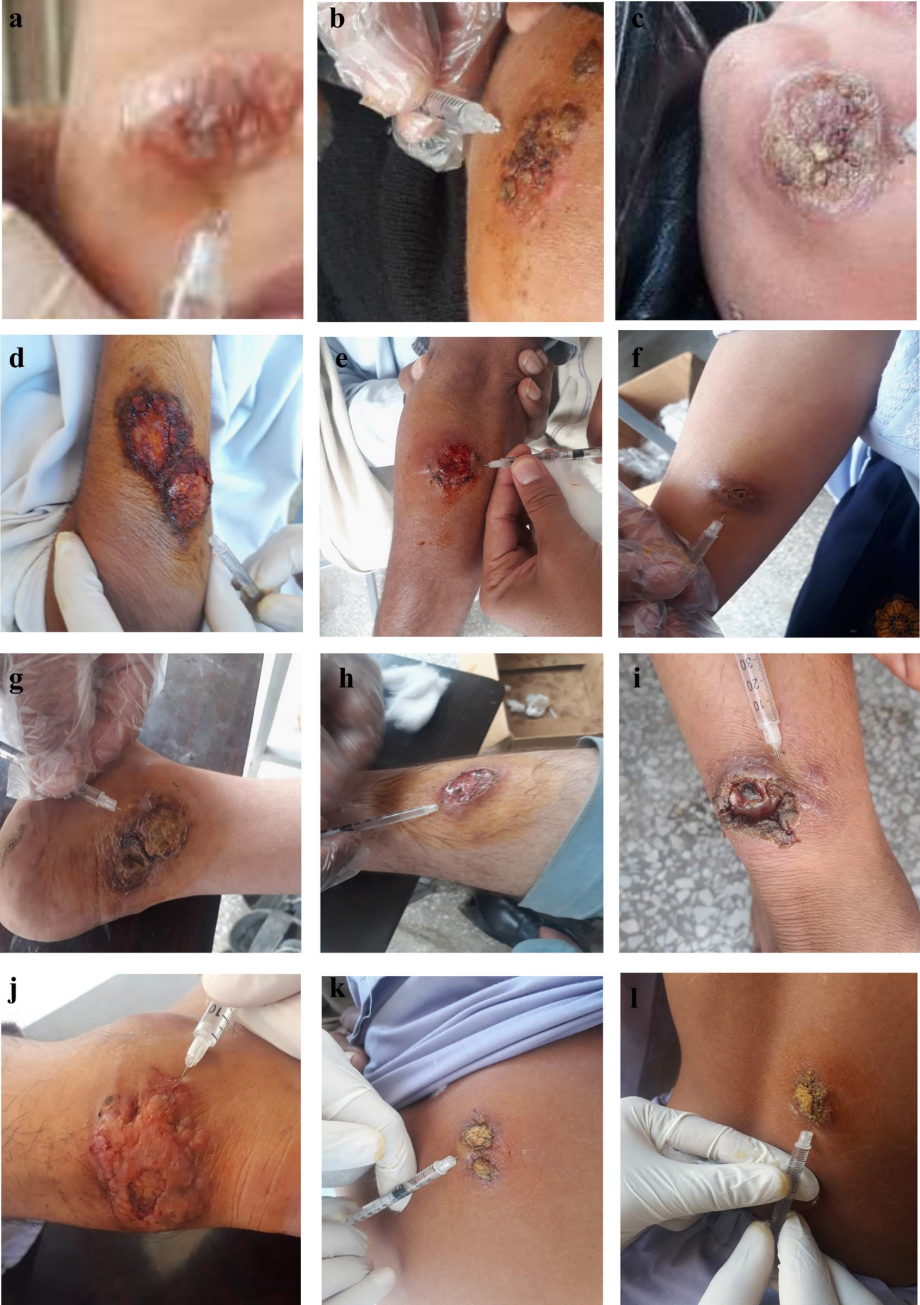
Skin lesions of cutaneous leishmaniasis on the face (a-c), hands (d-f), legs (g-j), and back (k, l) of participants.

Suspected individuals (patients) with skin lesions (**Fig 2)**, who visited dermatologist clinics and cutaneous leishmaniasis centres established in the study area, were recruited and a convenience sampling technique was used for data collection. Participants irrespective of their age, gender, education, occupation, language etc., were considered for this study. Sampling was based on visual inspection of an ulcerated skin/skin lesion and skin scraps were collected for diagnosis through microscopy. A disposable scalpel blade (no.11) was used to scrape the infected tissues and prepare a smear on a glass slide. All the procedures were ensured in the presence of medical representatives. A total of 1,674 suspected individuals were sampled during the study duration (May 2019 to December 2019).

### Questionnaire designing

A comprehensive questionnaire was designed after a detailed study of relevant literature and by following Frary’s guidelines for questionnaire construction [32]. The questionnaire was comprised of sections including sociodemographic characteristics, clinical features, knowledge, attitude, and practices of participants towards cutaneous leishmaniasis **(S1 File)**. Before data collection, the questionnaire was pre-tested and validated by circulating among experts and a small number of volunteers in the target population. Two researchers (MKO and MT) were trained and guided for field data sampling and the whole process was duly monitored/supervised by the principal investigators (SA and SH). An interpreter was also recruited if the participants were unable to understand the questionnaire. The proforma was duly filled and questionnaires of only confirmed individuals were further analysed.

### Microscopic examination

**Fig 3:**
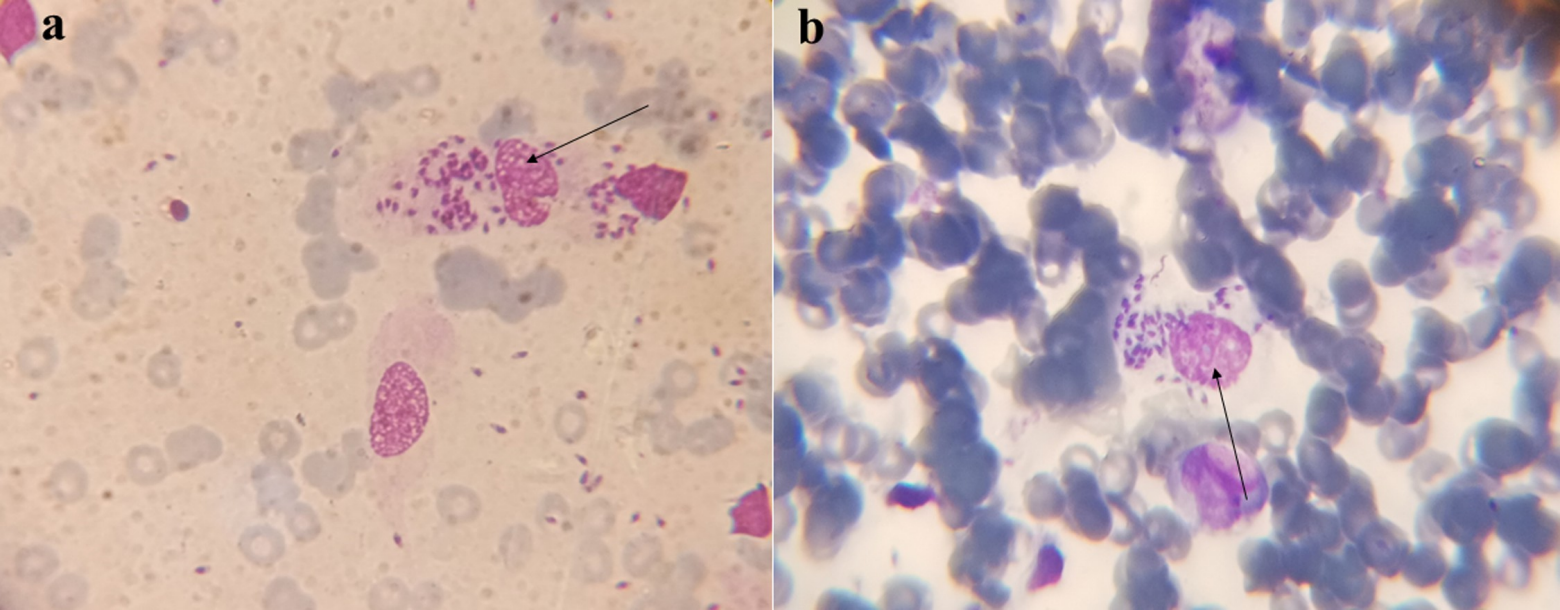
Arrows pointing to amastigote forms of *Leishmania* spp.

The dried smears of infected tissues (skin lesions) were processed according to the method performed by Bensoussan et al. [33] with slight modifications. Both thick and thin smears were prepared and stained with 5% Giemsa stain. The diagnosis was confirmed by observing amastigotes under the microscope (Optica, 500 series) **(Fig 3)**.

### Data analysis

Data were managed in Microsoft Excel and descriptive statistics were performed using IBM SPSS Statistics (Version 23) [26]. The prevalence of cutaneous leishmaniasis was calculated by using the following formula:

$$\text{Prevalence}=\text{Positive}\;\text{cases}/\text{total}\;\text{cases}\left(\text{positive}+\text{negative}\right)\times 100$$

## Results

**Fig 4:**
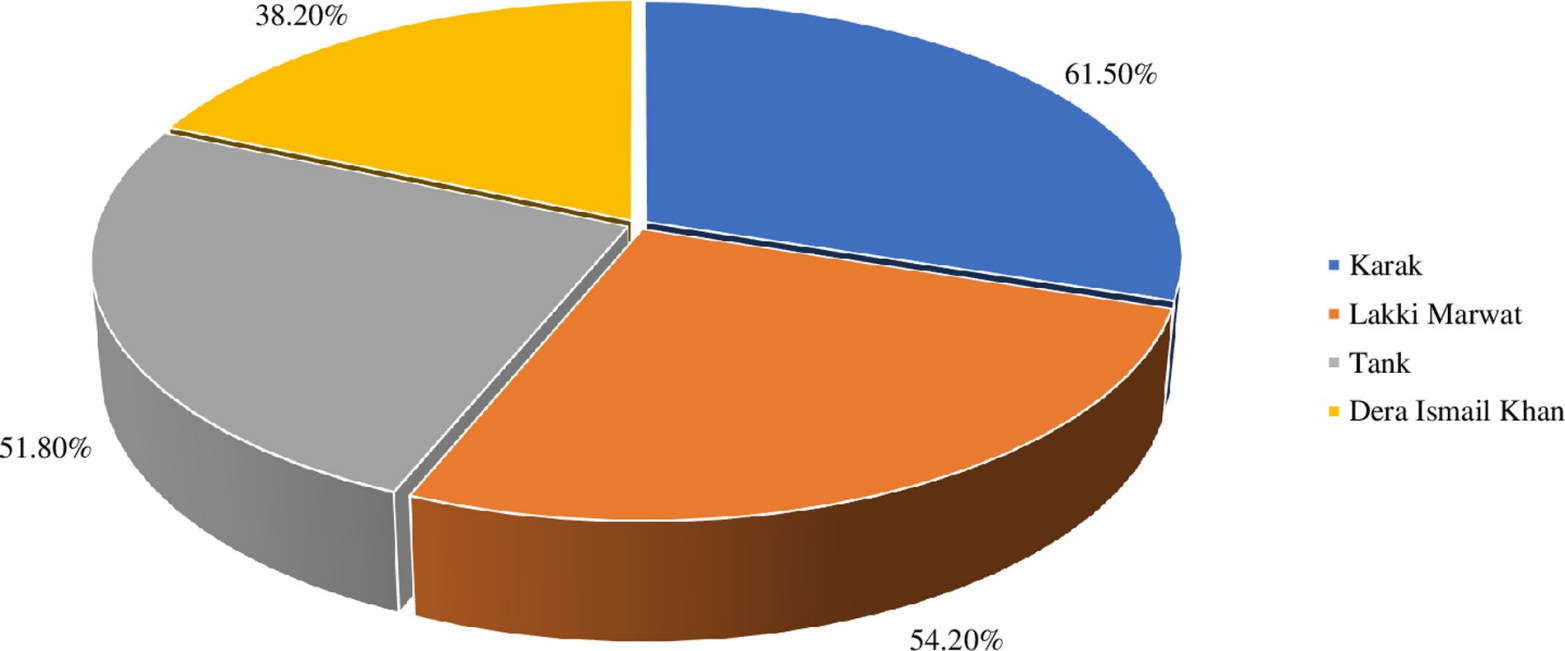
District-wise prevalence of cutaneous leishmaniasis in the study area.

A total of 1,674 suspected individuals participated in the current study and were subsequently interviewed and sampled to investigate the prevalence, knowledge, attitude, and practices about cutaneous leishmaniasis. Among the total participants, 844 were found positive for cutaneous leishmaniasis upon microscopy and hence, they were considered to determine the prevalence and further knowledge, attitude, and practices about the disease. The prevalence of cutaneous leishmaniasis was 50.4% and district wise the highest prevalence was recorded in Karak 61.5% followed by Lakki Marwat 54.2%, Tank 51.8%, and Dera Ismail Khan 38.2% **(Fig 4)**.

### Sociodemographic characteristics and clinical features of participants

Sociodemographic characteristics of participants having cutaneous leishmaniasis (n = 844) were analysed and are presented in **Table 1**. Most respondents 53.8% were observed to be in the age group ranging from 0 to ≤ 15-years, indicating the risk of infection decreases with age. Gender-wise distribution of respondents showed that 56.9% were male and 43.1% female. Most respondents 70.5% were of nuclear family while 29.5% were of extended family type.

**Table 1:** Sociodemographic characteristics of participants in the study area (n = 844).

Variables	Categories	Number	Percentage (%)
Age	0 to ≤ 15 years	454	53.8
	16–30 years	221	26.2
	31–45 years	86	10.2
	46–60 years	59	7.0
	≥ 60 years	24	2.80
Gender	Male	480	56.9
	Female	364	43.1
Position in the household	Head of the family	158	18.7
	Dependent member	686	81.3
Rural-Urban typology	Rural plain	354	41.9
	Urban plain	307	36.4
	Rural hilly	155	18.4
	Urban hilly	28	3.3
Family type	Nuclear	595	70.5
	Extended	249	29.5
Mother language	Pushto	692	81.9
	Saraiki	135	16.0
	Others	17	2.0
Education level in the household (≥ 5-years age (n = 793)	Illiterate	305	38.5
	Primary	276	34.8
	Middle	24	3.0
	SSC	123	15.5
	High School	49	6.2
	Graduation	16	2.0
Occupation (≥ 16-years age (n = 390)	Farmer	108	27.7
	Govt servant	72	18.5
	Driver	51	13.1
	Shopkeeper	55	14.1
	Labor	66	16.9
	Jobless	38	9.8
House type	Pucca	398	47.2
	Kutcha	446	52.8
The economic status of family	Low	460	54.5
	Middle	323	38.3
	High	61	7.2

Among the participants, 41.9% were inhabitants of rural-plain, followed by 36.4% rural-hilly, 18.4% urban-plain, and 3.3% urban-hilly areas. Additionally, 52.8% of respondents were living in kutcha houses while 47.2% were in pucca houses. The participants were mainly Pashto speakers 81.9% followed by Saraiki 16.0%, and other languages 2.0%. The ratio of illiterate participants (0 to ≥ 5-years) was higher 38.5%. Moreover, most participants 54.5% were from low-income families and most of them 27.7% were associated with farming **Table 1**.

Participants with multiple skin lesions 52.8% were more frequent than those with a single skin lesion 47.2%. The face was the most frequently affected body part 32.0% followed by the lower extremity 28.3%, upper extremity 27.5%, and lesions on multiple body sites 12.2%. Moreover, the ratio of participants having lesions older than one month was 80.3% and only 19.7% had a lesion in less than a month. Predominantly, 70.7% of the participants were infected with blood protozoan parasites for the first time **Table 2**.

**Table 2:** Clinical features of the study participants (n = 844).

Variables	Categories	Number	Percentage (%)
Number of the lesion(s) on the body of the participants	Single	398	47.2
	Multiple	446	52.8
Site of the lesion(s)	Upper extremity	232	27.5
	Lower extremity	239	28.3
	Neck and above	270	32.0
	Multiple sites	103	12.2
Duration of infection (appearance of the lesion)	≤ 30 days	166	19.7
	≥ 30 days	678	80.3
History of the participant with protozoan infections (if any)	Cutaneous leishmaniasis	4	0.4
	Malaria & toxoplasmosis	243	28.8
	Nil	597	70.7

### Knowledge, attitude, and practices of participants regarding cutaneous leishmaniasis

The knowledge and awareness of participants about the disease are presented in **Table 3**. Among the participants, only 35.2% had observed cutaneous leishmaniasis before attracting the disease. The majority 72.7% were unaware of the exact symptoms while 27.3% were knowing. Moreover, only 15.9% had basic knowledge about its vector. Similarly, the awareness about anthroponotic spread, preventive measures, secondary infections, and reservoir hosts was very low i.e., 2.8%, 2.0%, 1.7%, and 0.6%, respectively.

**Table 3:** Knowledge of participants about cutaneous leishmaniasis (n = 844).

Variables	Response	Number	Percentage (%)
Have you ever observed a person with cutaneous leishmaniasis before attracting the infection?	Yes	297	35.2
	No	547	64.8
Complete knowledge about the symptoms	Yes	230	27.3
	No	614	72.7
Awareness about the vector	Yes	134	15.9
	No	710	84.1
Awareness about the anthroponotic spread of cutaneous leishmaniasis	Yes	23	2.8
	No	821	97.8
Knowledge about the basic preventive measures of cutaneous leishmaniasis	Yes	17	2.0
	No	827	97.9
Knowledge about the chances of getting secondary infections and their prevention	Yes	15	1.7
	No	829	98.2
Knowledge about reservoir animals for cutaneous leishmaniasis	Yes	05	0.6
	No	839	99.4

The observed potential risk factors revealed that 52.6% of the respondents were using water from outdoor sources and hence, they were more exposed to sandflies’ bites. Most participants 77.0% were using organic fuel in the form of cattle dung or wood at home. About 96.0% of the participants were sleeping above and 4.0% were on the ground. Most of the respondents were indigenous residents of the districts’ understudy and only 3.8% were reported of having a migration history from other districts **Table 4**.

**Table 4:** Attitude and practices of participants towards cutaneous leishmaniasis (n = 844).

Variables	Categories	Number	Percentage (%)
Source of drinking water	Outdoor	446	52.8
	Indoor	398	47.2
Use of organic fuel (dung and wood) in the household for cooking and heating purposes	Yes	650	77.0
	No	194	23.0
Sleeping style of participant	Ground	34	4.0
	Above ground	810	96.0
History of migration from districts other than the study area	Yes	32	3.8
	No	812	96.2
Presence of animal reservoirs (wild) in close vicinity	Yes	838	99.2
	No	06	0.7
Animal reservoirs in close vicinity (frequently encountered on daily basis)	Cats	838	99.3
	Rodents	793	93.9
	Dogs	822	97.3
	Jackals	382	45.2
	Rabbits	293	34.7
	Bats	772	91.4
Domestic animals in the household of participants (cattle, canines, and birds)	Yes	819	97.0
	No	25	3.0
Use of mosquito nets while sleeping	Always	17	2.0
	Never	145	17.2
	Sometimes	682	80.8
Use of insecticide spray in the household	Always	512	60.6
	Never	124	14.7
	Sometimes	208	24.7
Use of mosquito repellents lotion/cream	Always	32	3.8
	Never	169	20.0
	Sometimes	643	76.2

Among the participants, 99.2% frequently encountered animal reservoirs in the wild and 97.0% were raising animals (domestic) at home. Comparatively, a trivial proportion of participants 2.0% were using mosquito nets, 3.8% were relying on mosquito repellents lotion/cream, and 60.6% were using insecticide sprays at home.

## Discussion

It is important to obtain an understanding of the community’s knowledge and practices along with the prevalence of any infectious disease in endemic areas to plan a control and management program effectively. The present study was a cross-sectional survey, aimed to assess the prevalence and knowledge, attitude, and practices among people with cutaneous lesions suspected of having cutaneous leishmaniasis in four districts of southern Khyber Pakhtunkhwa. The highest frequency of cutaneous leishmaniasis was mainly observed in children, which is in line with the study carried out by Hussain et al., 2018 [15]. Results showed a high occurrence of the infection in male individuals than in females. It is reported that males are more likely to attract the infection [34,35] because they are actively involved in outdoor activities. Furthermore, the male also prefers to sleep shirtless and outdoor without protective bed nets [36]. Another possible explanation could be that females remain covered most of the time because of religious and cultural standards in the study area [15]. However, some studies have also reported a high prevalence in female participants [16,21]. People living in kutcha houses were highly affected by the disease as compared to those living in pucca houses. The cracks and holes in mud walls may be serving as a shelter for sandflies in the study area. Previously, among household characteristics, mud-plastered walls were strongly associated with sandfly vector density [37]. Similarly, literacy levels have a strong association with the disease (awareness, transmission, prevention, etc.) as suggested by Razavinasab et al., 2019 [38] and in the current study, the highest infection rate was in illiterate participants.

The lesions were more common on the uncovered body parts i.e., the arms, face, and legs, which is very similar to previous reports [15]. Furthermore, most participants were with multiple lesions on their bodies as previously reported in district Karak and Multan [16,36]. Sometimes, the infected vectors may have difficulty taking blood meals due to a congested proboscis, consequently, several bites of the same host are required to acquire the optimum blood meal and hence, the number of lesions increases [36]. The participants were mainly from rural areas where risk factors such as the high number of domestic animals, unhygienic conditions, and poverty were predominant. Poor and unhygienic housing conditions may provide the best habitats for sandflies’ dwelling and propagation [39].

One of the main public health policies for cutaneous leishmaniasis management is to examine participants’ understanding of the infection, its prevention, and control methods. The knowledge and awareness of the participants were poor, and only a small proportion knew about the sandfly. Participants were mostly unfamiliar with the prevention, transmission, signs and symptoms, and reservoir hosts. Poor understanding of sandflies’ identification and control measures have also been reported in Punjab Pakistan [26]. A similar study conducted in Saudi Arabia found that only (37.4%) of participants could identify sandflies as the vector of cutaneous leishmaniasis [40]. Participants from endemic communities of Ghana were mostly unaware of the mode of transmission (80.2%) and preventive measures (39.6%) of the disease [41]. In contrast, Singh et al., 2006 from India confirmed a high level of awareness, in a rural community of Bihar state, about the biting time of sandflies, control, and preventive measures [42]. Communities’ awareness related to spreading, control, and prevention play a significant role in lowering the prevalence of cutaneous leishmaniasis [42].

Participants were found reluctant towards using bed nets and only a small proportion had been using bed nets as a preventive measure. In Waziristan, a large proportion of participants were reported to be using bed nets and insecticides as preventive measures against cutaneous leishmaniasis [27]. Health education can bring awareness to the community at risk and thus, can be helpful in the prevention of the disease at the individual and community levels [43]. Most participants were not aware of the sandfly as a vector for leishmaniasis and a similar trend was reported by Akram et al., 2015 [26] where 84.0% of the respondents were unable to identify the transmission of cutaneous leishmaniasis by the sandfly. People usually seem confused in differentiating sandflies from other common flies and mosquitoes. Reports from other countries have also revealed that most people were of the view that mosquitoes are responsible for the transmission [44,45]. Therefore, knowledge of the vector is significantly associated with the prevention and control of cutaneous leishmaniasis [46].

Most of the participants were unfamiliar with the signs and symptoms of cutaneous leishmaniasis and most of them had never been observed in any leishmaniasis patient. Similar trends of poor knowledge and understanding of the clinical manifestations of the disease have been reported in Pakistan [26,27] and India [45], where participants were unable to recognize pictures of cutaneous leishmaniasis patients. However, in Ethiopia, most participants were aware of the clinical manifestation of cutaneous leishmaniasis [44,47]. Pakistan is a developing country, and it is indispensable to educate and well inform the local communities about the risk factors, transmission patterns, and vectors of leishmaniasis. Unawareness of the disease emphasizes the need to initiate health education, awareness campaigns, and future in-depth research that will help to design applicable policies to guide government and stakeholders to reduce the risks of cutaneous leishmaniasis outbreaks in endemic areas [27].

### Limitations

Microscopic confirmation may compromise the actual prevalence of cutaneous leishmaniasis and thus, the molecular investigation would have provided more precise information on cutaneous leishmaniasis prevalence and the circulating *Leishmania* spp., [16] in the study area. However, unavailability of resources and funding were the major constraints of the current study and therefore, future in-depth studies evaluating not only *Leishmania* spp., but the sandfly vectors are highly recommended in these districts. In addition, the use of the convenience sampling technique was another limitation of the current study.

## Conclusions

An increase in the prevalence of the disease has been recorded and it is one of the major health problems in Khyber Pakhtunkhwa. Most participants were unaware of the basic knowledge of cutaneous leishmaniasis, its vector, preventive measures, control, transmission patterns, and major risk factors which can substantially contribute to the spread of infection among local communities. This study necessitates the planning and execution of regulations and preventive programs, public health education, awareness campaigns, and disease management practices to overcome future incidence of the infection.

## Supporting information

S1 File The questionnaire used for data collection related to cutaneous leishmaniasis. (DOCX)
