# Supplementary material for: Knowledge, attitude, and practices towards cutaneous leishmaniasis in referral cases with cutaneous lesions: A cross-sectional survey in remote districts of southern Khyber Pakhtunkhwa, Pakistan
Source: PLoS One. 2022 May 26;17(5):e0268801. doi: 10.1371/journal.pone.0268801 (PMC9135282; doi:10.1371/journal.pone.0268801)
Supplement: S1 File — (DOCX) [file pone.0268801.s001.docx]

**S1 File. The questionnaire used for data collection related to cutaneous leishmaniasis.**

**Questionnaire Form**

Name: _________________________ Father/Guardian name: ________________________

Address:_______________________________________________________________________________________________________________________________________________

Contact no: _________________

1. **Demographic features of participants**

- Age in years

i. 0-≤ 15 □ ii. 16-30 □

iii. 31-35 □ vi. 46-60 □

v. ≥60 □

- Gender

i. Male □ ii. Female □ iii. Other □

- Marital status

i. Married □ ii. Unmarried □ iii. Divorced □

- Education level

i. Primary □ ii. Middle □ iii. SSC □

iv. High school □ v. Graduation □ vi. Illiterate □

- Occupation

i. Farmer □ ii. Govt. Servant □ iii. Driver □

iv. Shopkeeper □ v. Labor □ vi. Jobless □

- Position in the household

i. Head of family □ ii. Dependent member □

- Place of birth

i. Lakki Marwat □ ii. Tank □ iii. D. I. Khan □

iv. Karak □ v. Other □

- Place of residence

i. Lakki Marwat □ ii. Tank □ iii. D. I. Khan □

iv. Karak □ v. Other □

- Rural-urban typology

i. Rural plain □ ii. Urban plain □ iii. Urban hilly □

iv. Rural hilly □

- Mother tongue

i. Pashto □ ii. Saraiki □ iii. Other □

- House type

i. Kuccha □ ii. Pucca □

- Family type

i. Nuclear □ ii. Extended □

- Economic status

i. Low □ ii. Middle □ iii. High □

# **B. Clinical features of cutaneous leishmaniasis**

# Number of the lesion(s) on the body

# i. Single □ ii. Multiple □

- Site of lesion

i. Upper extremity □ ii. Lower extremity □ iii. Neck and above □

iv. Multiple sites □

- Duration of infection (appearance of the lesion)

i. ≤30 □ ii. ≥30 □

- History of participant with protozoan infections (if any)

i. Cutaneous leishmaniasis □ ii. Malaria and toxoplasmosis □ iii. Nil □

# **C. Knowledge of participants**

- Have you ever observed a person with cutaneous leishmaniasis before attracting the infection?

i. Yes □ ii. No □

- Complete knowledge of signs/symptoms.

i. Yes □ ii. No □

- Awareness about the vector.

i. Yes □ ii. No □

- Awareness about the anthroponotic spread of cutaneous leishmaniasis

i. Yes □ ii. No □

- Knowledge about the basic preventive measures of cutaneous leishmaniasis

i. Yes □ ii. No □

- Knowledge about the chances of getting secondary infections and their prevention

i. Yes □ ii. No □

- Knowledge about animal reservoirs for cutaneous leishmaniasis

i. Yes □ ii. No □

# **D. Attitude and practices of participants**

- Source of drinking water

i. Outdoor □ ii. Indoor □

- Use of organic fuel (dung and wood) in the household for cooking and heating purposes.

i. Yes □ ii. No □

- Sleeping style of participant

i. Ground □ ii. Above ground □

- History of migration from districts other than the study area

i. Yes □ ii. No □

- Presence of animal reservoirs (wild) in close vicinity

i. Yes □ ii. No □

- Animal reservoirs in close vicinity (frequently encountered on daily basis)

i. Cats □ ii. Rodents □ iii. Dogs □ iv. Jackals □

v. Rabbits □ vi. Bats □

- Domestic animals in the household of participants (cattle, canines, and birds)

i. Yes ii. No □

- Use of mosquito nets while sleeping

i. Always □ ii. Never □ iii. Sometimes □

- Use of insecticide spray in the household

i. Always □ ii. Never □ iii. Sometimes □

- Use of mosquito repellents lotion/cream

i. Always □ ii. Never □ iii. Sometimes □
